# Temperature stabilization with Hebbian learning using an autonomous optoelectronic dendritic unit

**DOI:** 10.1007/s12200-025-00151-9

**Published:** 2025-04-03

**Authors:** Silvia Ortín, Moritz Pflüger, Apostolos Argyris

**Affiliations:** Instituto de Física Interdisciplinar y Sistemas Complejos, IFISC (UIB-CSIC), Ctra. de Valldemossa, km 7.5, Palma, 07122 Spain

**Keywords:** Optoelectronic system, Fiberoptic system, Input correlation learning, Neuro-inspired computing, Optical dendritic unit

## Abstract

**Abstract:**

The integration of machine learning with photonic and optoelectronic components is progressing rapidly, offering the potential for high-speed bio-inspired computing platforms. In this work, we employ an experimental fiber-based dendritic structure with adaptive plasticity for a learning-and-control virtual task. Specifically, we develop a closed-loop controller embedded in a single-mode fiber optical dendritic unit (ODU) that incorporates Hebbian learning principles, and we test it in a hypothetical temperature stabilization task. Our optoelectronic system operates at 1 GHz signaling and sampling rates and applies plasticity rules through the direct modulation of semiconductor optical amplifiers. Although the input correlation (ICO) learning rule we consider here is computed digitally from the experimental output of the optoelectronic system, this output is fed back into the plastic properties of the ODU physical substrate, enabling autonomous learning. In this specific configuration, we utilize only three plastic dendritic optical branches with exclusively positive weighting. We demonstrate that, despite variations in the physical system’s parameters, the application of the ICO learning rule effectively mitigates temperature disturbances, ensuring robust performance. These results encourage an all-hardware solution, where optimizing feedback loop speed and embedding the ICO rule will enable continuous stabilization, finalizing a real-time platform operating at up to 1 GHz.

**Graphic abstract:**

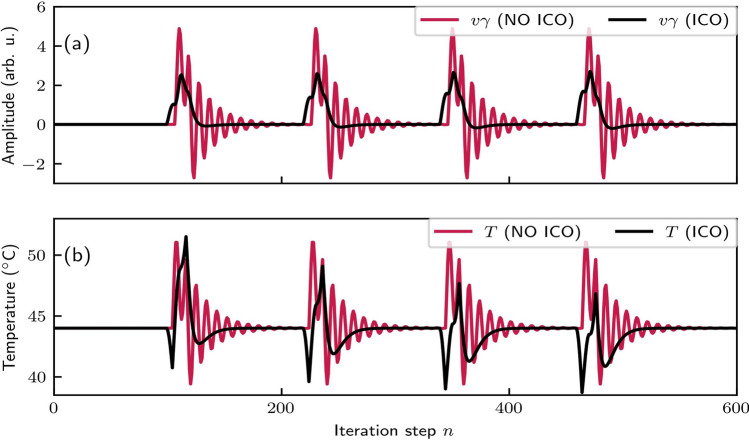

## Introduction

Temperature changes in physical systems typically occur over slow timescales when exposed to environmental conditions or when operating under active elements or processes that cause heating. The rate of these temperature changes is governed by the thermal properties of the materials involved and the heat transfer mechanisms at play. In many aspects of everyday life, active temperature control is efficiently implemented using PID (Proportional-Integral-Derivative) controllers in a closed-loop system [1], either with analog electronics or micro-controllers. The operational bandwidth of these controllers, up to a few kHz, is sufficient to manage slow-changing temperatures effectively. Modern PID controllers are often integrated with advanced control algorithms or tuning techniques, such as adaptive mechanisms [2], fuzzy logic [3], reinforcement learning [4] and neural networks [5], that optimize their performance in dynamic environments. However, there are many real-world scenarios in which temperature changes occur much more rapidly-within microseconds or even faster. Processes in laser-based thermal systems (such as laser ablation and laser annealing of thin films), semiconductor manufacturing, superconducting quantum systems, fusion reactors, high-speed electronics, and nanotechnology can induce localized heating followed by cooling at ultrafast timescales. These often lie in the kHz and up to the MHz range, where traditional PID controllers are inadequate. For such fast temperature fluctuations, precise and swift stabilization mechanisms are essential but challenging, due to the bandwidth and response time limitations of conventional electronic controllers. Digital ASIC-based and FPGA-based controllers are increasingly being used as potential solutions to address these challenges [6, 7].

On the other hand, recent advances in optoelectronic and photonic technologies have opened the door for their application in high-speed control tasks [8, 9]. Optoelectronic systems are known for their ability to operate at much higher frequencies than traditional electronic systems, reaching the GHz range. Optical components such as semiconductor optical amplifiers (SOAs), modulators, and photodetectors enable ultrafast processing of signals, which is critical for achieving the rapid response times required in fast temperature stabilization. Furthermore, optoelectronic systems offer inherent advantages such as low latency, high bandwidth, and immunity to electromagnetic interference, making them suitable for high-speed applications in dynamic thermal environments [10, 11]. Moreover, their capability to incorporate neuro-inspired concepts in their system designs, including synaptic plasticity, offers a promising alternative [12–15]. Hebbian learning, a foundational principle in biological systems, enables adaptive changes in synaptic strengths based on activity. This self-organizing principle can be applied to control tasks where the system adapts in real-time to environmental changes. In particular, input correlation (ICO) learning, a form of Hebbian learning tailored for control systems, allows continuous adaptation to dynamic conditions by adjusting control parameters based on correlated inputs [16, 17]. Such a paradigm shift from traditional control methods to learning-based approaches is highly advantageous for systems requiring ultrafast responses. Unlike PID controllers, which rely on predetermined control parameters, Hebbian and ICO learning-based systems can adjust and optimize in real time, making them ideal for environments with fast-changing conditions. These properties position optoelectronic systems as ideal platforms for implementing neuro-inspired control mechanisms like Hebbian learning.

In this study, we demonstrate an autonomously operating optoelectronic-based solution for fast temperature stabilization using an optical dendritic unit (ODU) inspired by biological neural networks. The ODU employs Hebbian learning to adapt to rapid temperature disturbances, providing a self-organizing mechanism for dynamic control. We integrate this system within a single-mode fiber (SMF) structure, where the temperature feedback is processed through three plastic dendritic branches (DBs). The hardware architecture, based on semiconductor optical amplifiers (SOAs) and capable of 1 GHz signaling and sampling, is capable of supporting MHz-scale temperature stabilization. The application of Hebbian plasticity rules, which are calculated via a PC for the time being, is enabled by direct modulation of the SOAs, further enhancing the system’s adaptability and responsiveness to rapid temperature changes. In our previous work [18], we presented an open-loop version of this system, demonstrating its potential for high-speed optical processing. Here, we extend that framework by introducing a closed-loop control mechanism that leverages Hebbian learning to dynamically adjust the system in response to temperature disturbances. In our demonstration, the proposed system can effectively counteract a repetitive temperature perturbation with minimal hardware complexity, using only three plastic DBs with positive weighting to modulate the temperature control output. This closed-loop architecture opens the path for real-time MHz bandwidth stabilization—via a subsequent hardware implementation of ICO learning—which is critical for applications where temperature fluctuations occur on ultrafast timescales.

## Temperature control with Hebbian learning

### ICO learning rule

**Fig. 1:**
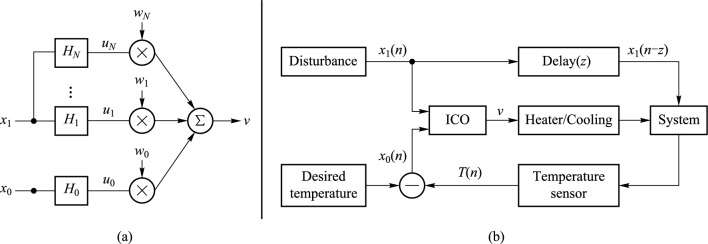
**a** Illustration of the ICO learning model for a temperature stabilization task. The tutor signal $x_0$ trains the synaptic weights $w_j$ ($j\ne 0$) in a heterosynaptic manner. **b** A temperature control system with ICO learning in a closed-loop configuration. The system is learning to keep the temperature constant against external disturbances. The temperature sensor establishes the feedback link from the ICO learning output *v* to the reference signal $x_0$

The ICO rule is a learning algorithm tailored for fast and stable learning in systems that depend on correlating inputs to adjust their behavior [17]. Unlike many conventional learning rules, which typically rely on feedback from the system’s output to adjust learning, ICO learning is driven solely by correlations among input signals. This input-focused mechanism allows ICO learning to adapt quickly and directly to environmental changes, leading to efficient, proactive adjustments without the delay that feedback-dependent methods often encounter. In ICO learning, the change of the input weight for the *j*th input ($j\ne 0$) is formalized as

1 $$\begin{aligned} \frac{\text{d}w_j}{\text{d}t} = \mu u_j \frac{\text{d}u_0}{\text{d}t}, \end{aligned}$$

where $w_j$ ($j\ne 0$) is the weight applied to input $u_j$, $\mu$ is the learning rate and $u_0$ is the reference (or tutor) input signal. $w_j$ ($j\ne 0$) is driven by the correlation between input $u_j$ and the derivative of the tutor signal $u_0$. The weight $w_0$, associated with the tutor input $u_0$, has a constant value. The input signals $u_j$ represent different filtered versions of a raw input *x*, which includes the sensory data received from the environment. The filtering process smooths the raw input, enabling the system to exploit meaningful temporal correlations between the signals. The way the signals are filtered depends on their role in the learning process. The raw reference signal $x_0$ is the guiding input for the ICO learner and is transformed into the filtered signal $u_0$ via:

2 $$\begin{aligned} u_0 = x_0 * H_0, \end{aligned}$$

where $H_0$ is a filter function. In a system that receives multiple raw inputs ($x_1$, $x_2$, etc.) the latter are processed through various filter functions, each capturing distinct temporal or frequency characteristics. In this work, we focus on an ICO system with only one raw sensory input $x_1$, apart from the tutor signal $x_0$ (Fig. 1a). The input $x_1$ passes through a set of $N-1$ filters $H_j$ ($j\ne 0$), producing different filtered versions $u_j$ of the same input $x_1$:

3 $$\begin{aligned} u_j = x_1 * H_j. \end{aligned}$$

The specific choice of filters $H_0$ and $H_j$ is not critical, as long as they meet the necessary conditions for ICO learning, namely introducing a low-pass characteristic and preserving temporal correlations, as described by [17]. While further fine-tuning may enhance performance, any filters satisfying these conditions will ensure stable learning and effective control.

Finally, the output *v* of the ICO system (Fig. 1a) is computed as the weighted sum of the filtered inputs $u_j$:

4 $$\begin{aligned} v = \sum _{j=0}^N w_j u_j. \end{aligned}$$

In a closed-loop feedback approach, the ICO learning rule works by sending the system’s output back to its sensory input, creating a continuous feedback loop [19, 20]. In this configuration, the system’s actions—driven by the output control variable *v*—alter the system environment, and these changes are sensed as new inputs. The ICO learning rule then uses this environmental feedback to adjust the system’s internal weights, enabling it to learn from its interactions with the environment. This process helps the system stabilize and reach a desired operational state.

### Temperature stabilization task

We apply the ICO learning rule within a temperature control system to stabilize the temperature within a desired range, despite external disturbances. In this framework (Fig. 1b), $x_1$ represents an external disturbance, such as a heat pulse, which affects the system temperature after a delay of *z* time steps. This delay enables the system to anticipate the disturbance and respond proactively. The reference input for the ICO learning rule, $x_0$, is defined as the error between the measured and the desired temperature. The temperature of the system at time step *n* is modeled as:

5 $$\begin{aligned} T(n) = T(n-1) - \gamma v(n) + \delta x_1(n-z), \end{aligned}$$

where $\gamma$ and $\delta$ are the coefficients that scale the impact of the control signal *v* and the external disturbance $x_1$ on the temperature, respectively. The parameter *z* represents the delay before the disturbance $x_1$ influences the system temperature. The ICO learning rule generates the control output *v*, which directly manages the heating ($v<0$) or cooling ($v>0$) processes to mitigate the external disturbance and maintain the target temperature. The primary objective is to minimize the error signal $x_0$. For instance, when a heat pulse ($x_1$) that leads to a temperature increase (reflected in $x_0$) is detected, the ICO learning rule adjusts the control weights so the system can begin cooling immediately, rather than waiting for the temperature to rise. By correlating the error signal $x_0$ with the disturbance $x_1$, the system learns to reduce errors in future events, effectively anticipating changes before they occur.

In the absence of the ICO learning mechanism, all plastic weights $w_j$ are constant or zero, and the control output *v* relies solely on the feedback signal. In that case, the system reacts only after a disturbance has caused a deviation from the target temperature $T_0$, adjusting *v* based on the error signal $x_0$. This reactive approach results in a delayed response, as the system can only adjust the temperature after detecting a change. The latter is an example of a standard PID controller, where the “weights” (typically the proportional, integral, and derivative gains) remain constant during the operation. These gains determine how the controller responds to errors but do not adapt dynamically in response to changing conditions. In conclusion, while the ICO learning rule facilitates a form of anticipatory control through correlation-based learning, the PID controller typically operates with a reactive control strategy based solely on existing errors.

Although this study focuses on temperature stabilization, the ICO learning rule and the optoelectronic dendritic unit can be adapted to other dynamic control tasks. The flexibility of the ICO rule in correlating error and alarm signals makes it suitable for various real-time adaptive control applications, provided appropriate signal definitions and parameter tuning are applied. For example, ICO learning has been used in mobile robot control, where the system learns to avoid obstacles by correlating an alarm signal from a proximity sensor with an error signal generated by a collision sensor [17]. Another example is in active noise cancellation systems [21], where the ICO rule contributes to the generation of an “anti-noise” signal.

## Autonomous single-mode fiber (SMF) optical dendritic unit (ODU)

**Fig. 2:**
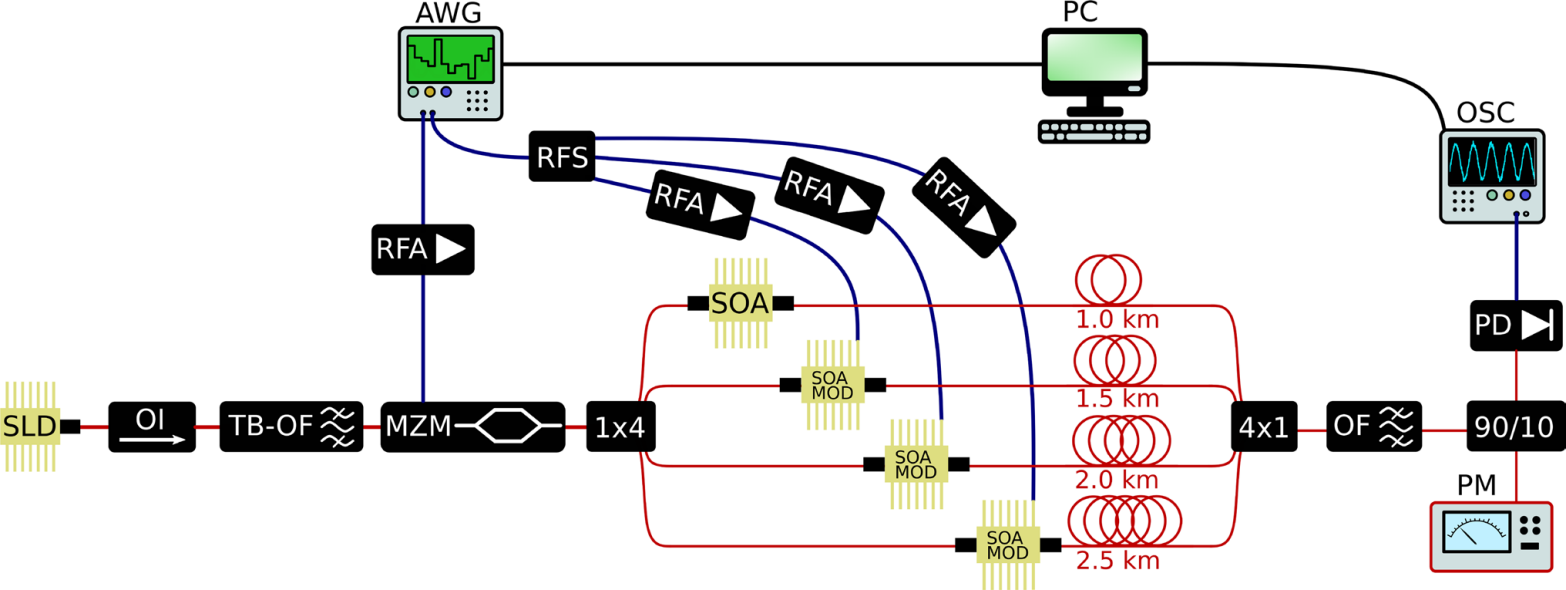
Red (blue) lines indicate optical (electrical) signals. The inputs $u_i$ and the weights $w_i$ are time-multiplexed in the arbitrary waveform generator (AWG) and physically demultiplexed in four different dendritic branches (DBs), by matching the time between different series generated by the AWG with the length of the SMF spools (1–2.5 km). The optical weights $w_{1,2,3}$ are calculated on a conventional computer (PC) from the output signal according to the ICO rule and applied from the AWG to the SOAMOD devices that perform the multiplication of $u_i$ with the plastic weight $w_i$. Since the first DB is used as the reference for the ICO learning rule, its weight is constant

In this work, we consider an optoelectronic system that applies the aforementioned ICO rule to a temperature stabilization task. A schematic of our experimental implementation based on a single-mode fiber optoelectronic dendritic unit (SMF ODU) with four DBs is shown in Fig. 2. We generate an incoherent optical carrier to the input of the ODU by combining a 40 mW superluminescent diode (SLD) at 1550 nm with an optical isolator and a tunable-bandwidth optical filter (TB-OF). Using the TB-OF, we limit the optical carrier’s bandwidth to 1.5 nm to suppress the signals’ chromatic dispersion while propagating within the ODU’s DBs. The input information ($u_0$ to $u_3$) is electrically encoded using a single channel of an arbitrary waveform generator (AWG) with a sampling rate of 10 GSa/s. The encoding is performed sequentially, via time-multiplexing. The four independent data streams that correspond to the individual filtered stimuli ($u_3$, $u_2$, $u_1$) and the reference signal ($u_0$) are combined into a single data stream in sequential order, separated by a temporal buffer to avoid any signal overlap in the responses from the system. This sequential encoded signal is then converted to the optical domain using a single 10 GHz Mach–Zehnder modulator (MZM) biased at its quadrature operating point. The modulated optical signal is split into four optical paths via a $1\times 4$ optical splitter, where each of the paths represents an optical DB. Each path includes a semiconductor optical amplifier (SOA), which can amplify or attenuate the optical signal. The three DBs that are considered for the experimental stimuli signals are dynamically weighted, following the adaptations required by the ICO rule. Thus, in these three DBs, the SOAs are operated as modulators (SOAMODs) by using the appropriate circuitry to modulate their bias current. The plasticity rules are introduced via electrical weight data streams ($w_3$, $w_2$, $w_1$), which are again combined into a single data stream in sequential order, using again time-multiplexing. These weighted sequences are generated using a second AWG channel. After electrical splitting (RFS), the corresponding signals are electrically amplified (RFA) and applied as current modulation to the SOAMOD devices. In the optical path of each DB of the ODU an SMF spool of increasing length (1, 1.5, 2, and 2.5 km) is included. This guarantees that the time-multiplexed signals encoded at the input can be temporally aligned at the coupling stage ($4\times 1$ optical coupler). The precision of the alignment is performed by shifting the signals at the encoding stage in the AWG, with an accuracy of 100 ps. At the incoherent summation stage of the ODU, we use a $4\times 1$ optical coupler that sums the optical signals from the DBs. An optical filter (OF) is used for the rejection of the amplified spontaneous emission noise introduced by SOA and SOAMOD devices. The power of the optical signal is monitored with an optical power meter (PM), and its temporal evolution is detected using a 20 GHz AC-coupled photoreceiver (PD). The converted electrical signal ($v^\textrm{E}$) is monitored and recorded using a real-time oscilloscope (OSC) with a 16 GHz analog bandwidth and a 40 GSa/s sampling rate. The oscilloscope’s high sampling rate is primarily used for fine-tuning and evaluating the temporal demultiplexing of the DB’s optical signals, rather than for capturing the signal itself, which has a lower bandwidth.

The above system describes the feed-forward optoelectronic operation of the SMF ODU. To introduce an autonomous operation with weight adaptation, we close the feedback loop by reading and processing the oscilloscope’s output response *v* with a conventional computer. Specifically, the electrical amplitude of the signal obtained at the oscilloscope, for a given iteration step, is used to calculate the plastic parameters of the ICO learning rule for the next step: the weights of the plastic DBs ($w_j$) and the reference input ($u_0$). These values are then updated in the encoding sequences at the AWG. While the optoelectronic system runs continuously and in real-time with a sampling rate of 10 GSa/s, the processing time needed for calculating the weights and communicating them via ethernet to the AWG’s interface is much longer (typically a few seconds).

While the current implementation computes the ICO learning rule on a PC and updates the system via Ethernet, this approach introduces a delay unsuitable for real-time high-speed tasks. Future improvements could involve hardware-based solutions, such as FPGAs or ASICs, which would eliminate communication delays and enable real-time processing at higher speeds. However, to implement a real-time operation of the ICO learning rule that effectively exploits the bandwidth of our optoelectronic system, we would need sensory elements that convert the error signal and the alarm signal into the optical domain. Moreover, the derivative function of the ICO rule (Eq. (1)) could be implemented with all-optical systems [22], and any background DC noise from the optical system could be eliminated considering a coherent optical system.

## Numerical study and parametrization of the system

**Fig. 3:**
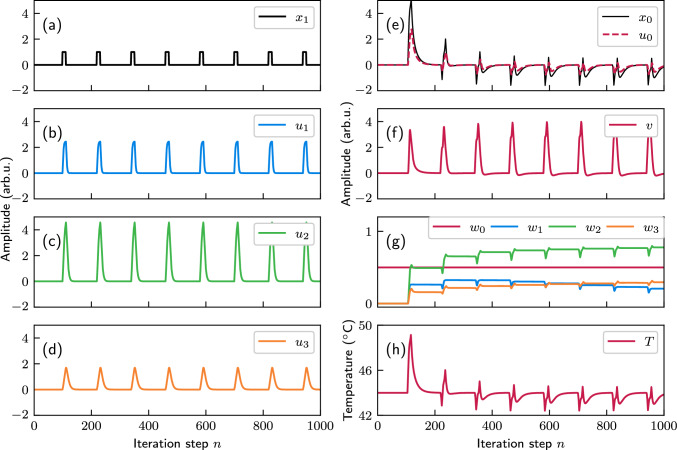
Results and input of the numerical simulation. **a** Periodic disturbance. **b**–**d** Filtered input signals, obtained from the periodic disturbance by filtering. **e** Evolution of unfiltered (red solid line) and filtered (blue dashed line) error signal. **f** Output signal. The system’s response depends largely on this signal. **g** Evolution of the weights. The weight $w_0 = 0.5$ is constant, while the other weights are updated according to the ICO rule. **h** Evolution of the system’s temperature. Due to ICO learning, the temperature spike caused by the third disturbance is significantly reduced compared to the first one

Before implementing the experimental ODU, we first conduct a numerical study of the system, introducing the signals that will be used. The primary objective is to define the structure of the signals and design a scenario in which a system with a hypothetical constant temperature is periodically disturbed by heat pulses. The disturbance signal $x_1$ is modeled as a periodic pulse with a normalized amplitude, lasting for 12 time steps, and repeated every 120 time steps, as shown in Fig. 3a. The system receives the predictive input $x_1$ from sensors that detect these heat pulses 6 time steps ($z = 6$) before they affect the temperature. In Eq. (5), we set $\delta = {0.8\,\mathrm{ ^{\circ }\text {C}}}$ to define the magnitude of $x_1$’s effect on the temperature, and $\gamma = {0.2\,\mathrm{ ^{\circ }\text {C}}}$ as the impact of the ICO rule output *v*. The desired temperature is fixed at 44 $^{\circ }\text {C}$ and the system is initialized in a stable state, where the current temperature equals the desired temperature, resulting in an initial error input $x_0 (0) = 0$. The plastic weights of the ICO rule ($w_3$, $w_2$, $w_1$) are set to zero, the fixed weight $w_0=0.5$, and the learning rate is fixed to 0.05. Before applying the ICO rule, both raw inputs $x_1$ and $x_0$ are filtered. The filter responses in discrete time *n* are described by:

6 $$\begin{aligned} H(n) = \frac{1}{bc}\text{e}^{an}\sin ({bn}), \end{aligned}$$

where $a=-\uppi f/Q$, $b=\sqrt{{(2\uppi f)}^2-a^2}$ and *c* controls the amplitude of the filtered signal. The parameter $0 \le f < 0.5$ is the normalized filter frequency, and $Q = 0.51$ defines the decay rate.

In our ODU configuration, we considered four DBs: one of them processes the reference filtered signal with a fixed weight $w_0$, and the other three branches correspond to different filtered stimulus signals $u_j$ with adaptive weights ($w_1$ to $w_3$). These filtered versions of $x_1$ are shown in Fig. 3b–d, and were obtained with filter responses with $f=0.1/j$ and $c=1,2,10$, for $j=1,2,3$ respectively. These inputs are independent of the system output and the present temperature *T*. The difference between *T* and the desired temperature $T_0$ defines the error signal $x_0(n)=T(n)-T_0$. This is the only input signal that depends on the control output and the system’s current temperature. Figure 3e shows the error signal $x_0$ and its filtered version $u_0$ obtained with a filter response with $f=0.1$ and $c=1$. The system runs autonomously, and the adaptive weights are updated according to the ICO learning rule (Eq. (1)) at each time step *n*. These updated weights (Fig. 3g) are then used to generate the control output *v*, as shown in Fig. 3f. Next, the control output *v* is applied to update the system temperature according to Eq. (5). The evolution of the system’s temperature (Fig. 3h) demonstrates that the system quickly learns to reduce temperature deviations caused by external disturbances. Actually, after the first disturbance pulse, the temperature oscillates in a range of 5 $^{\circ }\text {C}$ around the desired value, when after a few disturbance pulses it oscillates only within a range of 1.5 $^{\circ }\text {C}$. In the present configuration, the absence of any temperature compensation results in an increase in the system’s temperature, following the pattern of the periodic disturbances and the evolution described by Eq. (5).

**Fig. 4:**
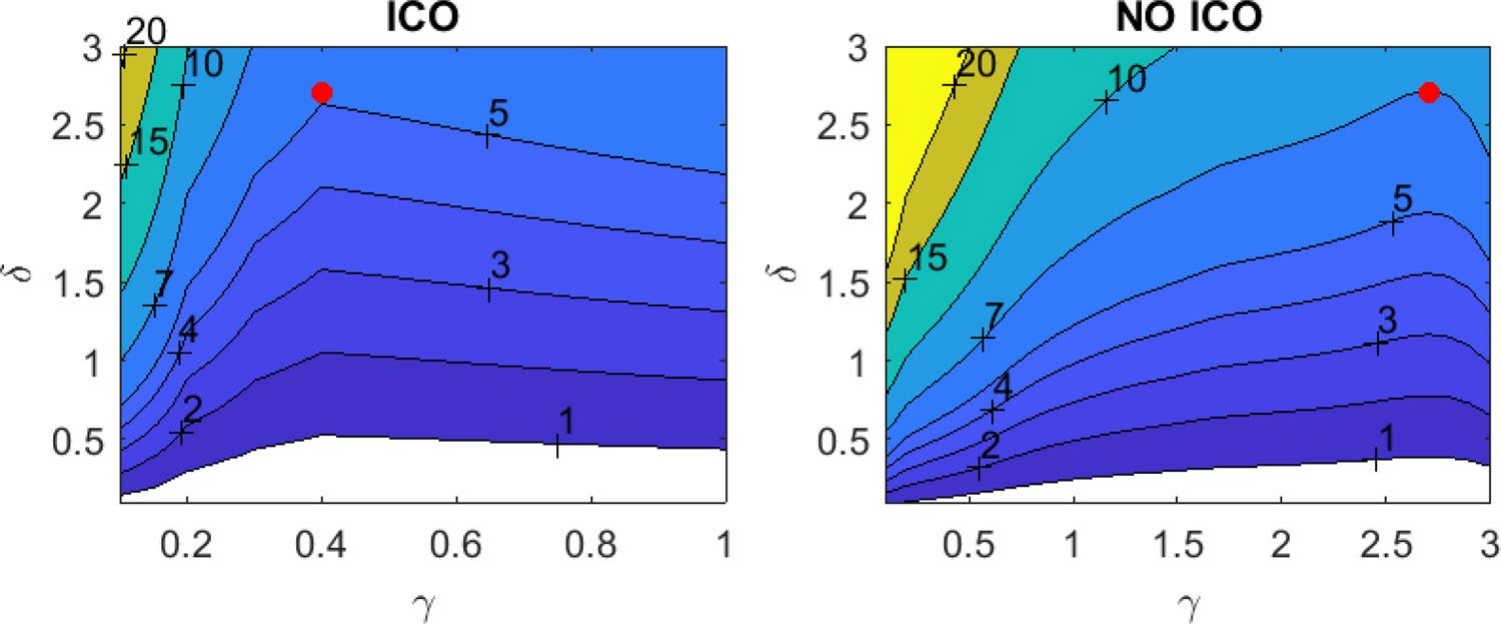
Temperature control performance with (left panel) and without (right panel) applying the ICO learning rule, versus the different scaling coefficients ($\gamma$, $\delta$) of the control signal and the external disturbance (Eq. (5)). The two panels display the maximum deviation in absolute value from the desired temperature of 44 $^{\circ }\text {C}$. In the “NO ICO” panel, the system responds to the disturbances without anticipatory control, while in the “ICO” panel, the system utilizes the predictive input from $x_1$

The left panel in Fig. 4 illustrates the effect of $\delta$ and $\gamma$ (Eq. (5)) on the temperature control task with ICO learning, showing the maximum absolute temperature deviation from the desired set-point of 44 $^{\circ }\text {C}$, after the system has stabilized. $\delta$ determines the strength of the disturbance $x_1$ on the temperature; larger $\delta$ values lead to higher temperature increases. $\gamma$ modulates the effect of the control output on temperature. In the left panel of Fig. 4, we observe that the optimal value for the $\gamma$ parameter is 0.4, when using the ICO learning rule. The right panel of Fig. 4 represents the case in which we do not apply any ICO learning rule. All weights are set to zero ($w_1=w_2=w_3=0$), and the system responds only to the feedback information received from the error signal $x_0$. In this case, the optimal value for $\gamma$ is 2.7. For the corresponding optimal values of $\gamma$ for the two cases, the ICO learning results in a maximum temperature deviation that is 1.4 times lower than the system without the ICO learning.

**Fig. 5:**
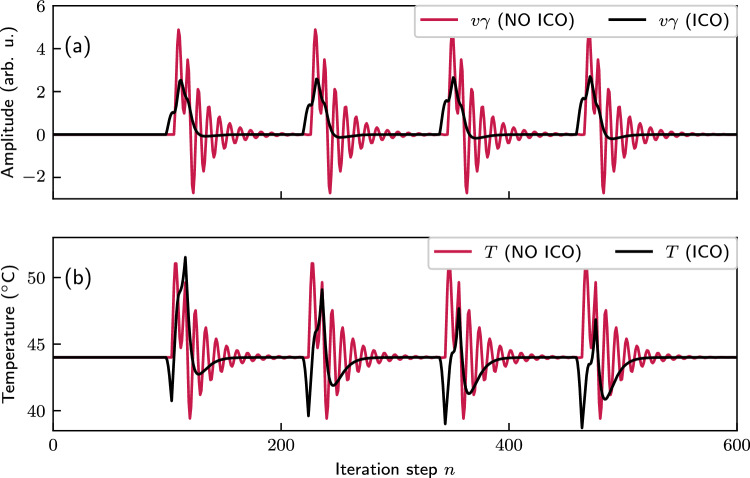
Comparison between the control task with (black lines) and without (red lines) ICO learning at their optimum $\gamma$ value, i.e., $\gamma =0.4$ and $\gamma =2.7$ for the ICO and no ICO case, respectively. $\delta =2.7$, the period of the disturbance is 120 and for the ICO rule the learning rate is 0.16. **a** The control output multiplied by the optimal $\gamma$ factor. **b** The system’s temperature for both cases

Additional insights into the advantages of ICO learning are provided in Fig. 5. Figure 5a shows the control output *v* multiplied by the optimal $\gamma$ for each case with $\delta =2.7$ (red points in Fig. 4). In a real-world system, this product would represent the strength of the heating or cooling. Figure 5b shows the resulting temperature response. The ICO learning configuration, by adjusting weights based on input correlations, can respond proactively, resulting in smoother, less intensive, and more stable heating and cooling cycles. This is especially beneficial in practical applications, as it reduces the complexity and energy demands associated with managing temperature fluctuations. Therefore, temperature control becomes more efficient and manageable, particularly in scenarios where rapid and adaptive temperature regulation is critical.

## Experimental closed-loop operation and comparison

In this section, we describe the experimental implementation, considering the input signals used in the numerical simulations while accounting for the constraints of the physical hardware. For example, only positive weights have been considered numerically, to follow the constraints of the electrical modulation that introduces the plasticity rules. The ODU experimental system is evaluated via the previously described temperature control task. The filtered inputs $u_1$, $u_2$, and $u_3$ (Fig. 3b–d) are generated numerically and are introduced to the AWG via time multiplexing, as explained in Sect. 3. The parameters used to simulate the temperature control system are the same as the ones used in the numerical simulations ($z=6$, $\delta =0.8$, and $\gamma =0.2$), and so are the initial conditions, i.e., $u_0(0) = w_1(0) = w_2(0) = w_3(0) = 0$, $w_0(0) = 0.5$. In the numerical study, the weights are normalized, while in the experimental implementation, they are physical quantities. The scaling of the electrical modulation is adjusted to exploit the dynamic range of the AWG and its encoding capabilities. This means that the lowest weight value, corresponding to zero for the normalized scale, is the lowest voltage provided by the AWG output ($-$0.5 V). Similarly, the highest weight value, corresponding to one for the normalized scale, is the highest voltage provided by the AWG output (+0.5 V). The physical electrical output of the AWG is then amplified or attenuated externally (RFAs in Fig. 2) to match the appropriate voltage scale required at the modulation input of the SOAMOD devices.

At the output of the ODU, the measured photodetected signal ($v^\textrm{E}$) needs to be preprocessed before it is used as a feedback signal for the autonomous operation. The rationale for that is that some physical quantities in the measured signal are affected by the measurement or the incoherent nature of the optical summation. In this preprocessing stage, the first step is to define and subtract any background DC noise from the oscilloscope ($c_0$), if other than zero. The second step is to scale the signal by a factor (*g*) to ensure that the amplitude of the experimental signal corresponds to the normalized one that we obtain from the numerical model. This is necessary since we need to convert a physically measured voltage value into a normalized value, which is then fed back into the system. Finally, in the last step, we need to account for the incoherent optical summation nature of the implemented ODU. The optical power that corresponds to the minimum encoding level of each weighted input pulse, at each DB, is always non-zero. This results in a weighted input for each DB of the form $w_i \cdot (u_i+d)$, instead of the form $w_i \cdot u_i$, where *d* is this non-zero bias term. To compensate for this, term *d* is multiplied by the sum of the experimental weights and subtracted from the normalized experimental output. The parameters *g* and *d* are obtained from the open-loop experimental system, without considering any feedback action and by fitting the output signal from the ODU to the simulated data. $c_0$ is determined directly as the average of the first 10 points of the oscilloscope output, where no signal has been introduced yet and is recalculated for each iteration step. All these corrections are introduced in the following equation, which provides the preprocessed experimental output signal ($v_\textrm{p}^\textrm{E}$) of the ODU at each iteration step *n*:

7 $$\begin{aligned} v_\textrm{p}^\textrm{E} (n) = g(v^\textrm{E}(n)-c_0)-d \sum _{i=1}^3 w_i^\textrm{E}(n), \end{aligned}$$

where $w_i^\textrm{E}$ are the weights obtained from the experimental closed-loop operation.

**Fig. 6:**
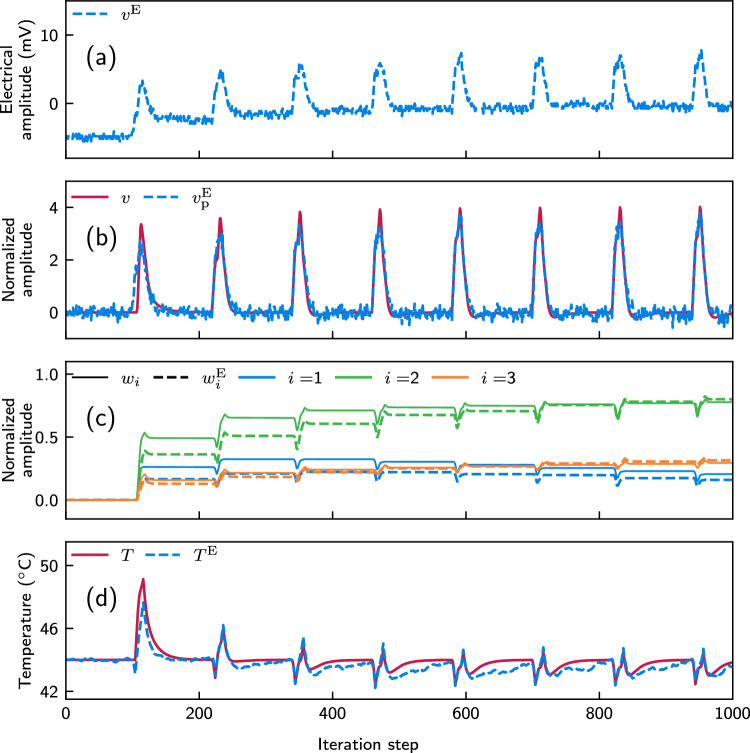
Temperature stabilization with ICO learning in an autonomous closed-loop ODU. The periodic external disturbance happens every 120 iteration steps. **a** Experimental photodetected ODU output ($v^\textrm{E}$) before pre-processing. **b** Pre-processed experimental photodetected ODU output (blue dashed line), according to Eq. (7). $g = 448/V$ and $d = 1.79$ lead to a fitted comparison with the numerical output signal *v* (red line). **c** Evolution of the three plastic DBs’ weights, when calculated numerically ($w_i$, solid lines) and when obtained experimentally ($w_i^\textrm{E}$, dashed lines). **d** Temperature evolution obtained from the numerical model (*T*, red solid line) and the experimental ODU ($T^\textrm{E}$, blue dashed line)

The description of the process for the experimental temperature control in an autonomous, iterative, and real-time manner is outlined below. With the previously specified initial conditions, the SMF ODU generates for the first epoch the first ODU output signal sample, which is photodetected and recorded with the oscilloscope. The electrical amplitude of this reading $v^E$ is then used to calculate the processed output ($v_p^\textrm{E}$) for this first iteration. Using this processed output, the plastic parameters of the ICO learning rule are calculated: the weights of the plastic DBs ($w_1$, $w_2$, and $w_3$) and the reference input ($u_0$). These updated weights, along with the filtered error signal, are then fed back into the SMF ODU to determine the properties of the next epoch in the sequence. This iterative process updates the plastic parameters in the AWG dataset for each new epoch, while the optical system continues operating continuously. As a result, the second sample of the ODU output signal is generated, photodetected, and recorded by the oscilloscope, and the process repeats. After 1000 iteration steps, we obtain a final electrical output signal from the ODU, reflecting the cumulative learning at each step (Fig. 6a). Every peak that appears every 120 iteration steps originates from the periodic disturbance that is applied at the given step. This learning incorporates preprocessing of previous outputs, parameterized in Eq. (7). To extract the relevant parameters of this equation, we fit the experimentally obtained output signal to the numerically derived signal. For $g = 448/V$ and $d = 1.79$, the processed output signal $v_\textrm{p}^\textrm{E}$, obtained from a closed-loop operation, closely aligns with *v*, the numerically generated output (Fig. 6b). However, slight deviations between the experimental and numerical results remain due to the incoherent optical summation and measurement noise. These small discrepancies affect the evolution of the weights and temperature control output. When comparing the evolution of the plastic weights for the increasing iteration steps (Fig. 6c), we see that they evolve similarly, but not identically. Despite these small differences in the evolution of the weights, the experimental closed-loop system demonstrates efficient temperature stabilization via the application of the ICO rule, as illustrated in Fig. 6d. In both cases, the temperature stabilization converges after a few learning cycles, reducing the impact of temperature disturbances significantly. Both systems eventually stabilize the temperature within a range around the target value of 44 $^{\circ }\text {C}$. In the experimental system, the temperature stays within the range from 42.4 $^{\circ }\text {C}$ to 44.6 $^{\circ }\text {C}$, while the numerical model predicts a slightly smaller temperature range between 42.5 $^{\circ }\text {C}$ and 44.5 $^{\circ }\text {C}$. This study demonstrates, both numerically and experimentally, how the system utilizing ICO learning becomes proactive, significantly mitigating the impact of periodic disturbances on its temperature. While the current experimental study focuses on moderate perturbations, increasing the scaling factor $\delta$ in the numerical model (Fig. 4a) indicates that temperature stabilization can still be achieved by proportionally adjusting the weights. However, the range of the stabilized temperature is increased when we consider larger $\delta$ values.

**Fig. 7:**
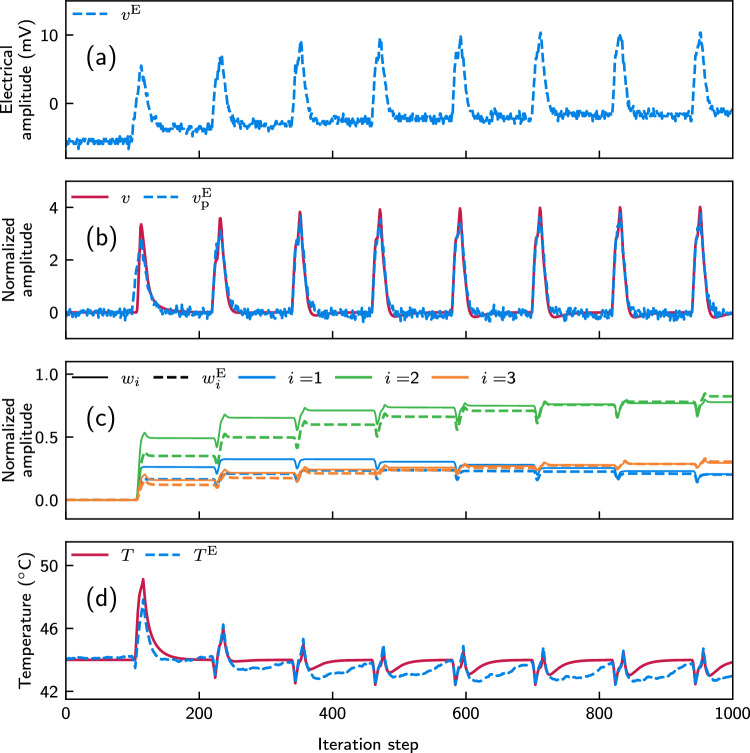
Same as Fig. 6, but for a different encoding configuration of our physical system that results in a different ODU output (**a**) compared to the case of Fig. 6, and for different pre-processing parametrization of the output signal, with $g = 287/V$ and $d = 1.05$ (**b**)

Additionally, the system exhibits robustness to variations in physical parameterization. As an example, we bias at a different operating point the MZM at the encoding stage of the ODU (Fig. 2), causing a different modulation index of the signals $u_j$. As shown in Fig. 7a, the obtained electrical amplitude at the ODU output is significantly larger than the one presented in Fig. 6a. Still, a similar evolution of the calculated experimental weights (Fig. 7c) and of the stabilized temperature (Fig. 7d) is obtained, by adjusting appropriately the pre-processing stage and it relevant parameters. In this example, these parameters are calculated to fit better the numerically obtained output (Fig. 7b) with the values $g = 287/V$ and $d = 1.05$.

## Conclusions

In conclusion, this study highlights the promising potential of an optoelectronic dendritic unit (ODU) with Hebbian learning for achieving temperature stabilization in dynamic environments. The integration of the ICO learning rule with an SMF-based ODU enables the system to autonomously adapt to temperature disturbances, offering an improvement over traditional PID controllers. This preemptive, steady response results in more stable temperature regulation, even under repetitive disturbances. Experimental validation, both through simulations and physical implementation, demonstrates the system’s capability for real-time dynamic adaptation. This work underscores the promise of neuro-inspired optoelectronic systems for real-time control applications, with implications for scalable and high-speed temperature regulation in environments demanding ultra-fast responsiveness. Future developments will focus on optimizing the learning feedback loop speed and integrating the ICO rule into hardware to further enhance continuous, high-frequency stabilization. These would be the last steps to convert the presented system into a real-time platform operating up to 1 GHz bandwidth.

## Data Availability

All data supporting the conclusion of this article will be made available by the authors, without undue reservation.

## References

[1] Ang, K.H., Chong, G., Li, Y.: PID control system analysis, design, and technology. IEEE Trans. Control Syst. Technol. **13**(4), 559–576 (2005)

[2] Radke, F., Isermann, R.: A parameter-adaptive PID-controller with stepwise parameter optimization. Automatica **23**(4), 449–457 (1987)

[3] Soyguder, S., Karakose, M., Alli, H.: Design and simulation of self-tuning PID-type fuzzy adaptive control for an expert HVAC system. Expert Syst. Appl. **36**(3), 4566–4573 (2009)

[4] Shuprajhaa, T., Sujit, S.K., Srinivasan, K.: Reinforcement learning based adaptive PID controller design for control of linear/nonlinear unstable processes. Appl. Soft Comput. J. **128**, 109450 (2022)

[5] Kumar, R., Srivastava, S., Gupta, J.: Artificial neural network based PID controller for online control of dynamical systems. In: 2016 IEEE 1st International Conference on Power Electronics, Intelligent Control and Energy Systems (ICPEICES), pp. 1–6 (2016)

[6] Kocur, M., Kozak, S., Dvorscak, B.: Design and implementation of FPGA-digital based PID controller. In: Proceedings of the 2014 15th International Carpathian Control Conference (ICCC), pp. 233–236 (2014)

[7] Sung, G.M., Chiang, P.Y., Tsai, Y.Y.: Predictive direct torque control ASIC with fuzzy voltage vector control and neural network PID speed controller. In: 2021 IEEE International Future Energy Electronics Conference (IFEEC), pp. 1–5 (2021)

[8] Gupta, M.C., Ballato, J.: The Handbook of Photonics. CRC Press, Boca Raton (2018)

[9] Dutta, N.K., Zhang, X.: Optoelectronic Devices. World Scientific, Singapore (2018)

[10] Udd, E., Benterou, J.: Improvements to high-speed monitoring of events in extreme environments using fiber bragg grating sensors. In: Fiber Optic Sensors and Applications IX, vol. 8370, pp. 155–167 (2012)

[11] Del Villar, I., Matias, I.R.: Optical Fibre Sensors: Fundamentals for Development of Optimized Devices. Wiley-IEEE Press, Piscataway (2020)

[12] Toole, R., Tait, A.N., De Lima, T.F., Nahmias, M.A., Shastri, B.J., Prucnal, P.R., Fok, M.P.: Photonic implementation of spike-timing-dependent plasticity and learning algorithms of biological neural systems. J. Lightwave Technol. **34**(2), 470–476 (2015)

[13] Feldmann, J., Youngblood, N., Wright, C.D., Bhaskaran, H., Pernice, W.H.: All-optical spiking neurosynaptic networks with self-learning capabilities. Nature **569**(7755), 208–214 (2019) 31068721 10.1038/s41586-019-1157-8 PMC6522354

[14] Huang, C., Sorger, V.J., Miscuglio, M., Al-Qadasi, M., Mukherjee, A., Lampe, L., Nichols, M., Tait, A.N., Lima, T., Marquez, B.A., et al.: Prospects and applications of photonic neural networks. Adv. Phys.: X. **7**(1), 1981155 (2022)

[15] Zheng, D., Xiang, S., Guo, X., Zhang, Y., Zeng, X., Zhu, X., Shi, Y., Chen, X., Hao, Y.: Full-function Pavlov associative learning photonic neural networks based on SOA and DFB-SA. APL Photonics **9**(2) (2024)

[16] Porr, B., Wörgötter, F.: Isotropic sequence order learning. Neural Comput. **15**(4), 831–864 (2003) 12689389 10.1162/08997660360581921

[17] Porr, B., Wörgötter, F.: Strongly improved stability and faster convergence of temporal sequence learning by using input correlations only. Neural Comput. **18**(6), 1380–1412 (2006) 16764508 10.1162/neco.2006.18.6.1380

[18] Ortín, S., Soriano, M.C., Tetzlaff, C., Wörgötter, F., Fischer, I., Mirasso, C.R., Argyris, A.: Implementation of input correlation learning with an optoelectronic dendritic unit. Front. Phys. **11**, 1112295 (2023)

[19] Kulvicius, T., Kolodziejski, C., Tamosiunaite, M., Porr, B., Wörgötter, F.: Behavioral analysis of differential Hebbian learning in closed-loop systems. Biol. Cybern. **103**, 255–271 (2010) 20556620 10.1007/s00422-010-0396-4

[20] Homchanthanakul, J., Manoonpong, P.: Continuous online adaptation of bioinspired adaptive neuroendocrine control for autonomous walking robots. IEEE Trans. Neural Netw. Learn. Syst. **33**(5), 1833–1845 (2022) 34669583 10.1109/TNNLS.2021.3119127

[21] Möller, K., Kappel, D., Tamosiunaite, M., Tetzlaff, C., Porr, B., Wörgötter, F.: Differential Hebbian learning with time-continuous signals for active noise reduction. PLoS ONE **17**(5), 0266679 (2022) 10.1371/journal.pone.0266679 PMC9135254 35617161

[22] Velanas, P., Bogris, A., Argyris, A., Syvridis, D.: High-speed all-optical first- and second-order differentiators based on cross-phase modulation in fibers. J. Lightwave Technol. **26**(18), 3269–3276 (2008)

